# Amyloid seeding as a disease mechanism and treatment target in transthyretin cardiac amyloidosis

**DOI:** 10.1007/s10741-022-10237-7

**Published:** 2022-04-06

**Authors:** Paolo Morfino, Alberto Aimo, Giorgia Panichella, Claudio Rapezzi, Michele Emdin

**Affiliations:** 1 Institute of Life Sciences, Scuola Superiore Sant Anna, Piazza Martiri della Libertà 33, 56124 Pisa, Italy; 2 Cardiology Division, Fondazione Toscana Gabriele Monasterio, Pisa, Italy; 3 Cardiologic Centre, University of Ferrara, Ferrara, Italy; 4 Maria Cecilia Hospital, GVM Care & Research, Cotignola (Ravenna), Ravenna, Italy

**Keywords:** Amyloidosis, Seeding, Heart, Therapy, Transthyretin

## Abstract

Transthyretin (TTR) is a tetrameric transport protein mainly synthesized by the liver and choroid plexus. ATTR amyloidosis is characterized by the misfolding of TTR monomers and their accumulation within tissues as amyloid fibres. Current therapeutic options rely on the blockade of TTR production, TTR stabilization to maintain the native structure of TTR, amyloid degradation, or induction of amyloid removal from tissues. “Amyloid seeds” are defined as small fibril fragments that induce amyloid precursors to assume a structure rich in β-sheets, thus promoting fibrillogenesis. Amyloid seeds are important to promote the amplification and spread of amyloid deposits. Further studies are needed to better understand the molecular structure of ATTR seeds (i.e. the characteristics of the most amyloidogenic species), and the conditions that promote the formation and multiplication of seeds in vivo. The pathological cascade may begin months to years before symptom onset, suggesting that seeds in tissues might potentially be used as biomarkers for the early disease stages. Inhibition of amyloid aggregation by anti-seeding peptides may represent a disease mechanism and treatment target in ATTR amyloidosis, with an additional benefit over current therapies.

Transthyretin amyloidosis (ATTR amyloidosis) is an increasingly recognized disorder due to population ageing and the introduction of non-invasive diagnostic algorithms [1]. ATTR amyloidosis is caused by the extracellular deposition of variant or wild-type transthyretin (TTR), which is a tetrameric protein mainly transporting thyroxine (${\mathrm{T}}_{4}$) and retinol in the plasma and brain. TTR amyloidogenesis occurs because of gene mutations or ageing-related alterations in the mechanisms of proteostasis, resulting in tetramer disassembly and misfolding of TTR monomers [2]. TTR monomers then self-assemble into fibrils with the peculiar cross-beta structure of amyloid substance. The most common forms of ATTR amyloidosis affect the heart and peripheral nervous system, leading primarily to cardiomyopathy and polyneuropathy [3]. The therapeutic approaches for ATTR amyloidosis are limited. Several pharmacological agents have been approved or are being tested and include drugs blocking TTR production, stabilizing the TTR tetramer or disrupting fibrils. The only approved drug for the treatment of isolated ATTR cardiomyopathy is tafamidis, a TTR stabilizer that seems less effective in patients with more advanced disease (New York Heart Association [NYHA] class III) [1]. Further investigations are then needed to better understand the pathophysiology of ATTR amyloidosis and to identify new therapeutic strategies.

Until few years ago, orthotopic liver transplantation (OLT) was the golden standard for the treatment of variant ATTR amyloidosis. However, liver transplantation often does not prevent disease progression. This led researchers to investigate the mechanisms behind disease progression in this setting, but also in other amyloid states (e.g. prion diseases). Many studies in vitro reported that amyloid TTR deposition occurs through a nucleation mechanism strongly correlated with the concentration of amyloid fibril fragments known as “seeds” [4, 5]. There is little evidence for nucleation in vivo, but the presence of truncated preformed fibrils might enhance and accelerate amyloid deposition within tissues. This process is known as “amyloid seeding,” and it is an effective mechanism of amplification and spread of amyloid aggregates [5]. Amyloid seeding represents also a possible therapeutic target. Halting TTR deposition by blocking the nuclei of ATTR seeds in tissues may then become an additive and complementary approach to tafamidis or other therapies to stop disease progression.

This review will focus on the current evidence on the pathogenic mechanisms and clinical relevance of the amyloid seeding phenomenon, as well as its inhibition as a potential therapeutic tool in ATTR amyloidosis. Pertinent studies were searched in PubMed/Medline (updated February 2022) using the following terms: “transthyretin amyloidosis”, “seeding”, “seed”, “Amyloid seeds”. Given the design of this work as a narrative review, no formal criteria for study selection or appraisal were enforced.

## Transthyretin amyloidosis: general concepts

### Transthyretin: structure and function

Transthyretin (TTR) is a homotetrameric transport protein mainly circulating in the plasma, cerebrospinal fluid and eye. TTR has a native globular structure with a ligand-binding hydrophobic channel at the centre of the tetramer, between the dimers [6, 7]. TTR monomers show many beta-sheet regions, resulting in a beta-sandwich structure [8], which helps explain the propension of TTR to form amyloid fibrils [7]. The subunits comprise 8 antiparallel beta-strands, assembled into a rigid core composed of two 4-stranded beta-sheets [9].

Circulating TTR is mostly synthesized by the liver and choroid plexus [10, 11]. TTR acts as a carrier of thyroid hormones (THs) and retinol-binding protein (RBP), this last bound to retinol (vitamin A) [12]. The binding stoichiometry of RBP and ${\mathrm{T}}_{4}$ enhances the structural stability of the TTR tetramer [13]. TTR stabilizers such as tafamidis mimic this mechanism. Conversely, many factors reduce TTR stability, such as oxidative modifications, aging, metal cations [14–16]. Finally, TTR is the pathogenetic agent of a form of amyloidosis.

### Epidemiology

ATTR amyloidosis derives from the accumulation of ATTR amyloid in the extracellular environment of different organs, especially the heart and peripheral nervous system [17]. Variant ATTR amyloidosis (ATTRv) is due to a single point mutation, with more than 130 pathogenic mutations described in the *TTR* gene [1]. Single mutations may destabilize the tetramer, promoting amyloid aggregation [18]. The most common variant associated with cardiomyopathy is pV142I, identified in USA and carried by 3–4% of African Americans [19, 20]. Instead, pV50M is the most frequent pathogenetic variant in patients with polyneuropathy, with a prevalence of 1:538 in northern Portugal and 4% in northern Sweden, while the carrier frequency in the Swedish population is about 2% [20–22]. The average age at disease onset is 39, and the course is variable according to the relative severity of cardiac or neurological involvement [23]. In wild-type ATTR (ATTRwt) amyloidosis, the *TTR* gene sequence is preserved but TTR may become kinetically unstable due to a general aging-associated decrease in protein quality control mechanisms. Ageing is indeed associated with a dysregulation of proteostasis, such as proteasome activity and unfolded protein response system, but also with an impairment in the bio-energetic efficiency and age-related changes in the extracellular matrix (ECM) composition and the extracellular space volume [18, 24–28]. In ATTRwt amyloidosis mainly affects the heart and most commonly elderly subjects. In a large cohort of 12,400 subjects undergoing ^99m^Tc-3,3-diphosphono-1,2-propanodicarboxylic acid (^99m^Tc-DPD) scintigraphy for different reasons, 45 (0.36%) were diagnosed with ATTR cardiac amyloidosis [29]. More than 90% of patients with ATTRwt amyloidosis are men and Caucasian, with an average age at diagnosis of 74 years [30]. Recently, the apparent incidence of ATTRwt-related cardiomyopathy has dramatically increased, mostly because of greater disease awareness and the introduction of an algorithm for non-invasive diagnosis [31].

### Clinical presentation

In ATTRv amyloidosis, the specific mutation determines the preferential involvement of the heart or peripheral nervous system, or more often a mixed phenotype, whereas ATTRwt amyloidosis affects mostly the heart, frequently leading to isolated cardiomyopathy. Until recently, cardiac amyloidosis (CA) was an under-recognized cause of heart failure, especially with preserved ejection fraction [18].The large amyloid deposits in myocardium result in the loss of physiological tissue architecture and function. Patients with ATTR-CA show progressive biventricular wall thickening without ventricular dilatation, causing a restrictive cardiomyopathy and low to normal cardiac output [32]. Diastolic dysfunction and atrial wall infiltration promote atrial fibrillation, and amyloid infiltration of the conduction system may cause conduction disorders [33].

### Prognosis

The clinical course in ATTRv amyloidosis is more variable than in ATTRwt. Untreated patients with pV142I ATTRv amyloidosis have a mean survival of 2.5 years, and those with ATTRwt of 3.6 years [34–37]. In early-onset ATTRv-V30M amyloidosis death usually occurs after a median of 12 years [38]. Late-onset ATTRv-V30M amyloidosis and ATTRv caused by other *TTR* mutations often display a more severe course, with a median survival of about 7 years [39, 40]. The Tafamidis in Transthyretin Cardiomyopathy Clinical Trial (ATTR-ACT) studied the safety and tolerability of tafamidis compared to placebo in both patients with ATTRwt and ATTRv amyloidosis, during a follow-up of 30 months [41]. Patients on placebo had an all-cause mortality of 43% and a cardiovascular mortality of 36%, both lower in ATTRwt amyloidosis, while both ATTRwt and ATTRv placebo-treated patients showed a hospitalization rate of 60%. Kansas City Cardiomyopathy Questionnaire Overall Summary Score decreased by 3.5 points every 6 months in ATTRwt amyloidosis and by 5.5 points in ATTRv. Therefore, ATTR amyloidosis is a slowly progressing disease, and its advanced state causes greatly decreased functional capacity, with high mortality and frequent hospitalization [18, 42].

### Therapies 

Several treatments have been proposed for the treatment of ATTR amyloidosis [17]. These approaches include blockade of TTR production, TTR tetramer stabilization and anti-seeding strategies [1].

OLT aims at preventing TTRv production and prolong survival [43]. As stated above, amyloid deposition in tissues, including heart and nerves, may continue after OLT [44], reasonably because of amyloid seeding. Data from an autopsy study reported that patients revealed different TTR concentrations in the heart before and after OLT (60% TTRv and 40% TTRwt vs. 25% TTRv and 75% TTRwt), highlighting the ongoing TTRwt deposition [44–47]. OLT is not indicated for patients with ATTRwt amyloidosis, because we would replace a source of ATTRwt with another source of ATTRwt, in a setting where amyloid deposition in the heart is enhanced by amyloid seeds.

Several small-interfering RNA (siRNA) and antisense oligonucleotides (ASOs) have been designed to silence *TTR* gene expression (either variant or wt). First-generation therapies include patisiran and inotersen [1]. Patisiran was approved by the FDA and European Medicines Agency (EMA) for the treatment of ATTRv amyloidosis with polyneuropathy [17], and inotersen was approved for the treatment of patients with mild or moderate (stages 1 and 2) ATTRv-related polyneuropathy [17]. Second-generation therapies include the siRNA vutrisiran, the ASO AKCEA-TTR-LRx and a new CRISPR-Cas9-based gene-editing in vivo approach (NTLA-2001).

The TTR stabilizer tafamidis was approved in 2011 by the FDA and EMA as the first disease-modifying therapy for adult patients with stage 1 polyneuropathy. Both FDA and EMA then approved tafamidis for the treatment of ATTR-CA [17, 41]. AG10 has a greater affinity for TTR than tafamidis and achieves near-complete TTR stabilization in patients with ATTR-CA [48], but has a shorter half-life [49].

Anti-seeding molecules such as TabFH2 inhibit TTR aggregation catalysed by preformed amyloid fibrils [4] and could hypothetically be combined with other therapies such as tafamidis or patisiran or stop disease progression in patients undergoing OLT. The following sections will cover the mechanism of seeding and the perspectives for its inhibition in ATTR amyloidosis.

## The amyloidogenic cascade

### Structure of amyloid fibrils

Amyloid fibres are long, unbranched structures formed by the polymerization of hundreds to thousands of monomeric peptides. The fibres are typically 5–15 nm in width and several micrometres in length and bind the Congo red and thioflavin T dyes [50].

The fibril is the fundamental structure of all amyloid fibres. Some amyloid fibrils are composed of a single protofilament, but the majority are formed by multiple subunits twisted together [51]. Each protofilament is a pile of protein layers with a beta-sheet structure, composed of beta-strands connected laterally [20]. The aggregation of monomers into protofilaments starts with the assembly of momentarily or permanently misfolded proteins into oligomers [52]. The rearrangement to form beta-strands starts from amyloid prone regions [53]. This organization named cross-beta resembles a sort of ladder made of beta-strands oriented perpendicularly to the fibril axis, with hydrogen bonds between consecutive beta-strands [51, 54, 55]. Interactions that stabilize the amyloid fibres include interdigitated hydrophobic or polar side chains [53, 56, 57]. The result is a very stable structure, both thermodynamically and mechanically. In Alzheimer’s disease (AD), oligomers are considered the main source of damage, and it has been suggested that the fibrillar deposits may be protective rather than pathogenic [58].

### TTR fibrils

TTR tetramer dissociation is the starting point and the rate-limiting step in amyloid fibril formation [59–63]. The TTRwt tetramer is thermodynamically more stable than misfolded monomers. Mutations in the CD loop of the protein may have a stabilizing (e.g. pT119M) or destabilizing (e.g. pS52P) effect on the tetramer. In particular, pathological mutations shift the equilibrium towards the monomer state and protective mutations towards the tetramer form. Therefore, unfolding mechanisms may originate at CD loop strands, and the loss of native quaternary structure quickly leads to the dissociation into unfolded monomers [64].

Studies assessing the self-assembly process of TTR have identified the F and H beta-strands as necessary for TTR aggregation [65]. These beta-strands are normally buried in the tetramer, but exposed in the monomer. Experiments of proline substitution within each segment allowed to identify the F beta-strands as an aggregation-driving segment. A similar experiment of substitution of ${\mathrm{Thr}}^{119}$ and ${\mathrm{Val}}^{121}$ to tyrosine and tryptophan, respectively, pointed to the beta-strand H is an aggregation-driving segment [65]. Single mutations in these strands lead to their exposure and to increase the rate of tetramer dissociation.

**Fig. 1:**
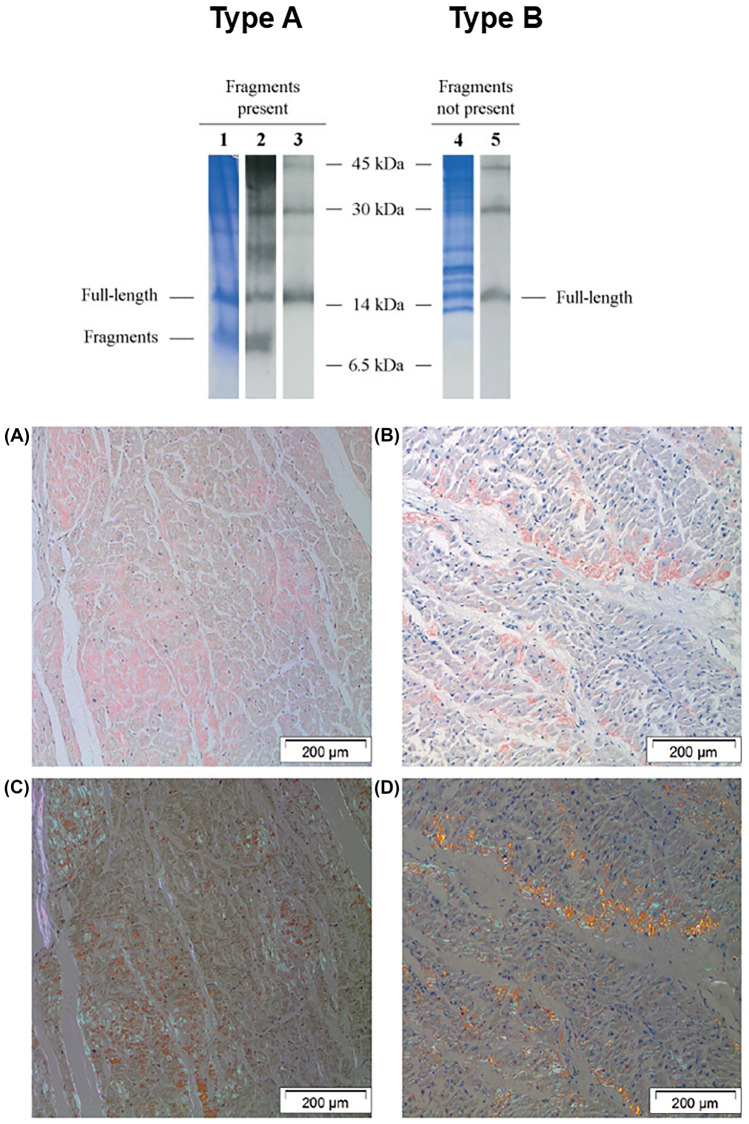
Type (**A**) and type (**B**) fibres. *Above:* Analysis of amyloid constituents in abdominal adipose tissue. Lanes 1–3 show the result from a patient in whom both fragmented and full-length ATTR was detected (pattern **A**), while lanes 4 and 5 show the result from a patient in whom only full-length ATTR is seen (pattern **B**). Lanes 1 and 4, Coomassie blue–stained SDS–polyacrylamide gels; lanes 2 and 5, Western blots using antiserum against TTR50-127; this antiserum reacts with full-length ATTR as well as the C-terminal fragments; lane 3, Western blot using commercial antibody against transthyretin (Dako); this antibody does not detect the truncated ATTR. *Below:* red staining of cardiac tissue with amyloid of the two fibril types. (**A**) and (**C**) a typical case of type A amyloid, showing faint staining with Congo red and an equally faint and smooth birefringence in polarized light. (**B**) and (**D**) a typical case of type (**B**) amyloid, showing strong staining with Congo red and a strong and granular birefringence in polarized light, creating a ‘‘glittering’’ effect. Reprinted with permission from: Ihse et al. [69]; Ihse et al. [70]

During fibrillogenesis, TTR initially forms a dimer through interactions between monomers, then these dimers aggregate into a hexamer with a spherical structure [66]. The existence of two types of TTR amyloid fibrils has been proposed: type A amyloid fibrils are formed by a mixture of both C-terminal fragments and full-length TTR, whereas type B amyloid fibrils by full-length TTR only [2]. Type A and B fibres have been associated with different patterns upon Congo red staining (Fig. 1). Truncated forms derive from proteolytic processing of the amyloidogenic precursor, both ATTRv and ATTRwt, but the identity and location of the protease(s) responsible for the cleavage and whether the cleavage occurs before or after fibrillogenesis have not been established yet [66]. Cryo-electron microscopy (cryo-EM) data showed that type A Val30Met ATTR amyloid fibrils contain TTR truncated forms derived from mutant and wild-type protein [67]. Patients with type A fibrils in their cardiac deposits have a remarkably worse prognosis due to a high proportion of truncated species, which promote and accelerate amyloid seeded-propagation [68].

### Amyloid formation

The kinetic of amyloid formation is shared by different forms of amyloidosis and includes a lag (or nucleation) phase, an elongation (or growth) phase and a plateau (or saturation) phase [51]. The first step is the formation of a nucleus, which is the smallest aggregate stable enough to grow through monomer addition. This process occurs in the lag phase and is also defined as primary nucleation [20]. Interestingly, the lag phase may be shortened or even eliminated by adding preformed seeds (seeding phenomenon) [71]. Since primary nucleation is a stochastic process, it occurs only at a certain critical concentration, temperature and peptide-length below which amyloid formation is impossible [71]. Non-fibrillar oligomer formation occurs as well, which may be on or off the fibril forming pathway [31]. In both murine models of ATTRwt deposition and human FAP, non-fibrillar aggregates precede the appearance of fibrils in the same location as the subsequent fibrillar deposits [72, 73].

The nucleation phase is followed by the elongation phase. Monomer aggregation is promoted because of the reduction in free energy due to the formation of chemical bonds that stabilize the compound [71]. The process continues with a sigmoidal kinetics until the formation of amyloid fibrils and then fibres. A dynamic equilibrium exists between different molecular species whereby fibrils can fragment and release new toxic oligomeric terminations that induce the recruitment of other monomers and the formation of new fibrils [74, 75]. While amyloid formation is a thermodynamically favourable process, it is very slow and might require years to occur spontaneously in vitro, suggesting that catalysing factors may accelerate the process in vivo [51]. There are still major differences between in vitro and in vivo models of fibrillogenesis, mainly because of the hard reproducibility in experimental models of amyloid disease.

Amyloid fibrils may also assemble with each other, with other proteins such SAP, or with extracellular matrix components [76–79]. Amyloid fibres and their precursors, especially oligomers, may damage cell membranes and activate the mechanisms of apoptosis and also cause a mechanical disruption of tissue architecture [51].

## Amyloid seeding

### Amyloid seeds

The common property of systemic amyloidoses is the process of amyloid diffusion, which may occur through a seeded mechanism in vivo [38]. “Amyloid seeds” are small fibrillary fragments that induce amyloid precursors to assume a much more stable structure rich in beta-sheets, thus promoting fibrillogenesis. Amyloid seeds are deemed important mechanisms of amyloid progression [80, 81].

Ranlov first recognized the relevance of seeding as a pathogenic mechanism in 1960s, followed by Kisilevsky et al. who theorized the “amyloid enhancing factor” as a transmissible element prompting amyloid deposition [82, 83]. It has also been demonstrated that tissues extracted from brains of AD patients induce amyloid Aβ aggregation in vitro [84]. The role of seeds was better understood in prion diseases (e.g. Creutzfeldt-Jakob disease, Kuru, fatal insomnia), where misfolded isoform seeds (e.g. ${\mathbf{P}\mathbf{r}\mathbf{P}}^{\mathbf{S}\mathbf{c}}$) induce naive proteins (e.g. ${\mathbf{P}\mathbf{r}\mathbf{P}}^{\mathbf{c}}$) to assume a similar pathogenic structure [85–88]. For a long time, prions were considered different from other amyloid proteins because of their “infectious” property. Nonetheless, the notion of a seed-mediated corruption of otherwise healthy proteins has been extended to amyloidosis [82, 89]. It is now believed that many amyloid diseases may spread both within and outside of an individual through a seed-dependent mechanism, leading to the notion of “transmissible amyloidoses”, such as AA amyloidosis [81]. The fibril protein AA derives from serum amyloid A (SAA), and it is a consequence of a severe and sustained inflammatory condition resulting in the overproduction of SAA [38]. The time to develop amyloidosis is greatly reduced if organ extracts of an amyloidotic donor are injected into a recipient with chronic inflammation [38, 90]. The presence of amyloid seeds in vivo is suggested especially by indirect evidence, such as the ATTR amyloidosis progression after OLT and the similarity between the course of disease progression and the kinetics of amyloid formation. Direct evidence of amyloid seeds is represented by their visualization in tissues through electron microscopy [91].

### Clinical relevance of amyloid seeding

**Fig. 2:**
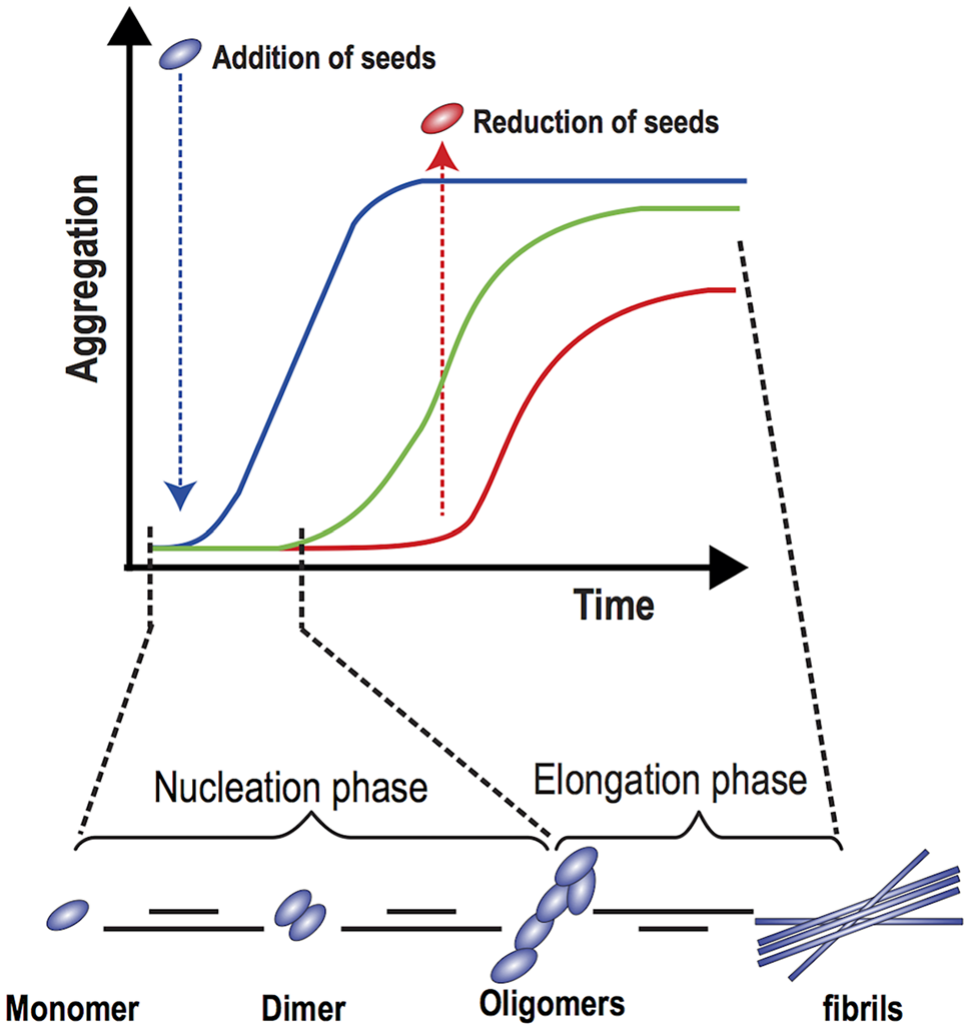
Amyloid aggregation in the presence of seeds. See text for details. Modified with permission from: Dayeh et al. [93]

Seeding has great clinical relevance because it is a crucial determinant of tissue amyloid deposition. Indeed, once amyloidogenesis starts, it becomes a self-propagating process through the generation and amplification of new seeds. The sigmoid kinetics of deposition of amyloid aggregates is a function of the rates of nucleation growth and fibrils fragmentation [80, 91, 92] (Fig. 2). The initial slow phase may be shortened or even abolished by adding preformed seeds to the system, which rapidly drive protein aggregation into fibrils [80]. Given that each fibril releases new seeds, the greater is the amyloid tissue content, the faster is further amyloid deposition.

Although OLT suppresses TTRv production and should therefore stop disease development, progression of cardiac and neurological involvement despite OLT has been frequently reported, with evidence of tissue deposition of wtTTR amyloid [28, 94]. One of the predictors of better outcome after OLT is a short disease duration, which is related to a lower amount of amyloid in tissues [95]. Other potential prognostic factors may be age, disease stage, specific mutation and tissue deposition patterns. Notably, mechanisms other than seeding cannot be excluded to explain disease progression, such as inflammation following tissue damage, although the inflammatory response to ATTR accumulation is remarkably low.

In domino liver transplantation, transplantation of a liver from donors with ATTRv amyloidosis may lead to the development of de novo amyloidosis in recipients after a quite short lag phase [96]. The most reasonable explanation might be the transmission of amyloid seeds through the liver, which trigger amyloid formation, although this point is debated [38]. Indeed, systemic TTR deposition may also depend on a lower proteostatic capacity of the liver related to age, long-standing exposure to misfolded TTR, or both [97].

Interestingly, amyloid seeding may explain why patients with amyloid TTR type A fibrils have a greater tendency to recruit TTRwt leading to disease development or progression, compared to patients with TTR type B fibrils. Indeed, type A fibrils may release a greater number of amyloid seeds [28]. As stated above, we might also postulate that the greater efficacy of tafamidis in less symptomatic patients (NYHA class I-II vs. III) is derived, at least partially, by a lower extent of amyloid deposits, with less amyloid seeds counteracting the therapeutic effect of tafamidis [23].

Amyloid deposition may be promoted not only by similar seeds, but by seeds composed of different proteins [98]. This phenomenon is named “cross-seeding” and may partly explain the possible finding of different amyloidogenic proteins in some disorders (e.g. AD) [99]. On the other hand, there is also evidence of “cross-inhibition” of fibrillogenesis, with TTR inhibiting Aβ fibril formation both in vitro and in transgenic models of human AD [100–102]. Our understanding of cross-seeding and cross-inhibition is currently limited [99].

### Effects of ATTR seeds and their inhibition

**Fig. 3:**
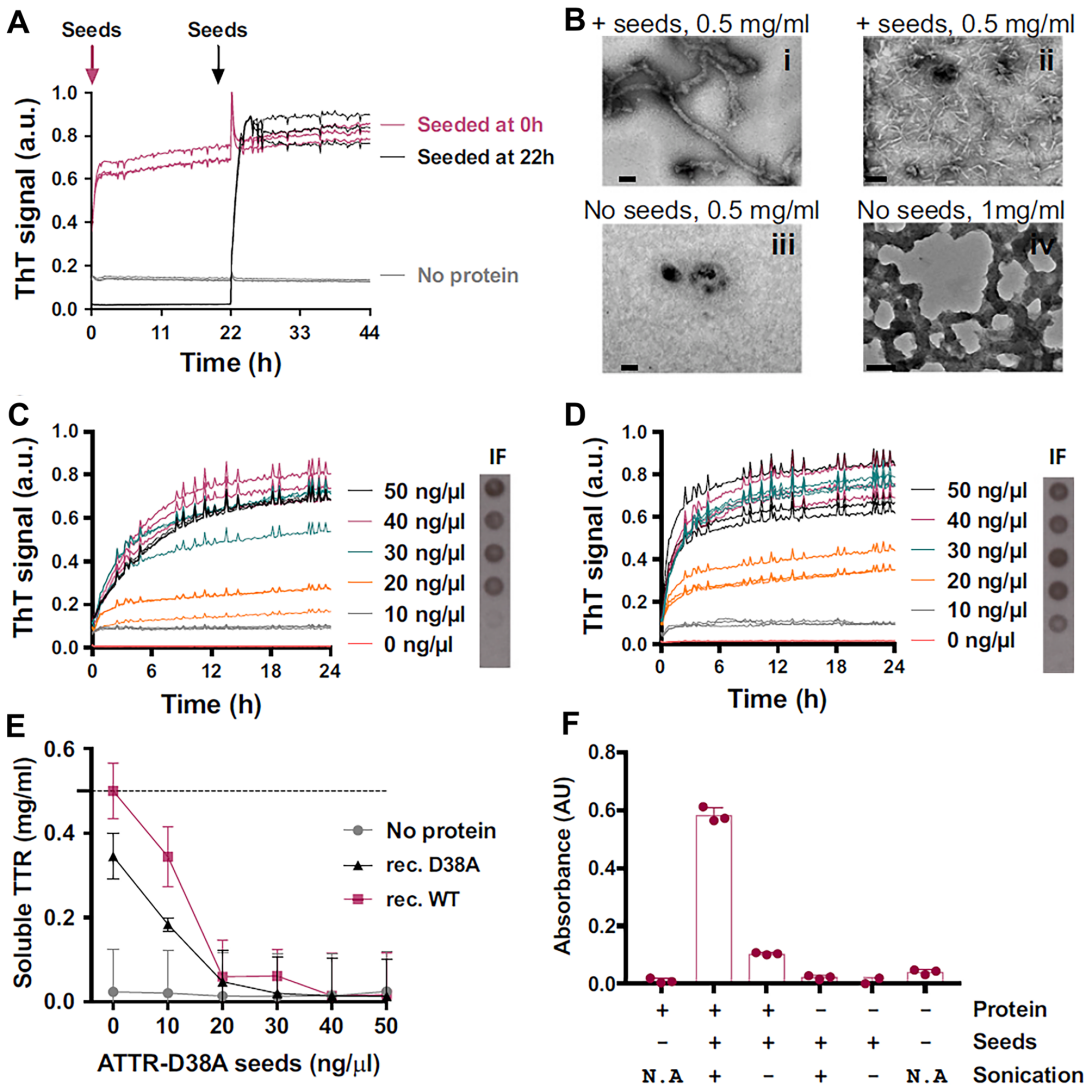
Effects of amyloid transthyretin (ATTR) seeds in vitro. Amyloid seeding at pH 4.3 of wild-type TTR by ex vivo ATTR seeds extracted from the explanted heart of an ATTR-D38A patient. (**A**) Amyloid seeding assay of recombinant wild-type TTR when 30 ng/μL ATTR-D38A ex vivo seeds were added at 0 h and after 22 h of preincubation, as monitored by thioflavin T (ThT) fluorescence. (**B**) Electron micrograph of aggregates of wild-type TTR after 24 h (i and iii) or 4 days (ii and iv) of incubation. ATTR seeds were added at 0 h (i and ii). (**C** and **D**) Amyloid seeding assays monitored by ThT fluorescence. Increasing amounts of ATTR-D38A ex vivo seeds were added at time 0 to recombinant wild-type TTR (**C**) or D38A TTR (**D**). (**E**) Protein concentration in the soluble fraction extracted from (**C** and **D**). Increasing concentrations of D38A seeds promote less soluble TTR due to TTR dissociation. The dashed line marks the initial protein concentration (0.5 mg/mL). (**F**) The 280-nm absorbance of insoluble fractions collected from an amyloid seeding assay of recombinant wild-type TTR with and without 30 ng/L sonicated or non-sonicated seeds. AU, absorbance units; a.u., arbitrary units; N/A, not applicable. Reprinted with permission from: Saelices et al. [5]

In vitro studies reported that ATTR seeds can promote and accelerate fibril formation, and there is clinical evidence of ATTR seeding in vivo [103]. Saelices et al. demonstrated that amyloid seeds extracted from ATTR tissues induces the formation of TTRwt or TTRv fibrils in vitro under acidic conditions (pH = 4.3) [5] (Fig. 3). Indeed, the acidic pH induces TTR tetramer dissociation, and then the addition of seeds promotes the aggregation of TTR monomers in a dose-dependent manner [5]. ATTR seeds do not induce amyloid aggregation of TTRwt under physiological conditions (pH = 7.4), while they prompt amyloid formation of engineered monomeric TTRv, carrying the double mutation F87M-L110M, even at pH = 7.4 [104]. These results further confirm that tetramer dissociation into monomers is a necessary step for amyloidogenesis even in the presence of seeds.

The sonication of ATTR fibrils greatly enhances their seeding capacity by causing fibril fragmentation [5]. Sonication enabled researchers to obtain higher concentrations of fibril fragments in vitro. The seeding capacity is strongly correlated with the concentration of C-terminal fragments, which are abundant in patients with type A amyloid fibrils [5]. Conversely, a comparison assay of amyloid seeding capacity and the amount of prefibrillary species reported that seeding capacity does not correlate with other parameters such as age in vitro [5]. However, the actual physical nature of seeds has always posed a problem, and fibrillar deposits in vivo are the result of in vivo selection for stability which is never achieved in the time frame of in vitro fibril formation experiments. However, studies conducted in mice transgenic for human wtTTR and V30M-TTR reported that in contrast to the AA and ApoAII amyloidoses, in vivo seeding may not occur for TTR under physiological condition. The injection of seeds possibly does not normally promote fibril formation because tetramer dissociation is the rate limiting step for amyloidogenesis [105, 106].

### Seeding inhibitors as therapies for ATTR amyloidosis 

Anti-seeding therapies may prove particularly effective in patients undergoing an OLT for ATTRv amyloidosis or in patients with an advanced disease stage, when the large number of seeds within tissues is likely to limit the efficacy of other therapies such as TTR stabilizers, as explained above. However, other explanations for treatment failure in patients with longstanding disease include the early initiation of tissue damaging processes in vivo that become self-sustaining even after the fibrils are removed. The recent experience with anti-Aβ therapy in human AD in which the deposits are largely mobilized by antibodies (aducanumab, donanemab), as determined by PET scanning, yet the clinical response is minimal, suggesting the fibrillar deposits are not necessarily responsible for the functional deficits once the disease is well established [107].

**Fig. 4:**
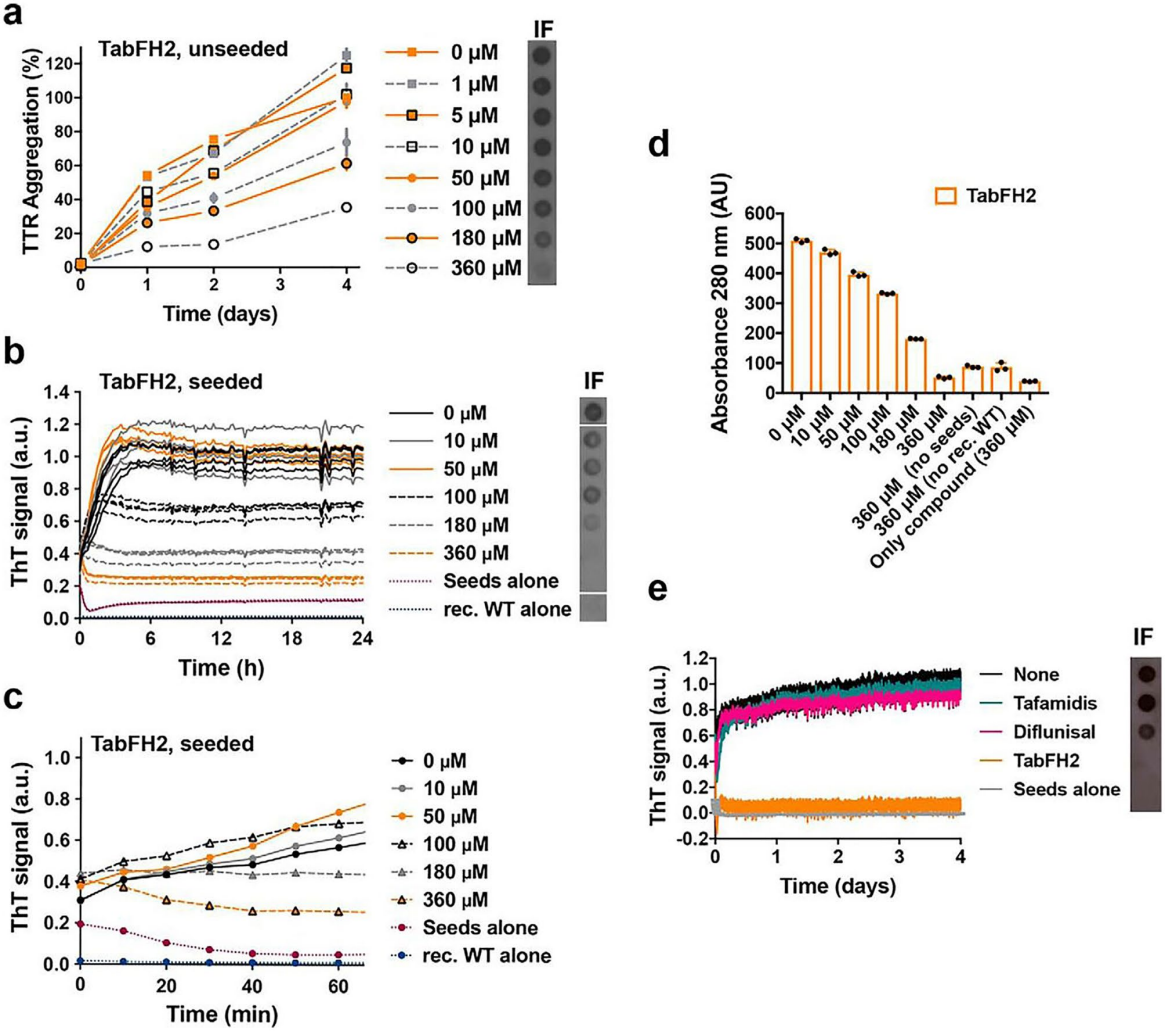
The anti-amyloid peptide inhibitor TabFH2 blocks transthyretin (TTR) aggregation and amyloid seeding caused by ATTR ex vivo seeds extracted from ATTR-D38A cardiac tissue. **a** Inhibition of TTR aggregation by TabFH2 in the absence of seeds, measured by absorbance at 400 nm. Increasing amounts of TabFH2 were added to 1 mg/ml of recombinant wild-type TTR and the sample was incubated for 4 days at pH 4.3. **b** Inhibition of amyloid seeding by TabFH2 at pH 4.3, monitored by ThT fluorescence. Increasing amounts of TabFH2 were added to 0.5 mg/ml of recombinant WT TTR and 30 ng/L of ATTR-D38A seeds. **c** Short-time view of the lag phase of the assay shown in **b**. **d** Protein content quantification of the insoluble fractions collected from b, measured by 280-nm absorbance. **e** Comparison of inhibition of amyloid seeding by tafamidis, diflunisal, and TabFH2 when incubated for 4 days, measured by ThT fluorescence. Reprinted with permission from: Saelices et al. [4]

The compound named TabFH2 is a mixture of two peptides (TabF2 and TabH2) which efficiently cap the amyloidogenic segments of TTR by binding the F and H β-strands of TTR, which are important segments driving aggregation [64]. “Tab” stands for “transthyretin aggregation blocker”, and the number 2 refers to the second version of the peptide [5]. In vitro studies reported that TabFH2 inhibits TTR aggregation by amyloid seeds in a dose-dependent manner, with complete blockade at higher doses [4] (Fig. 4). The efficacy of TabFH2 clearly displays an inverse correlation with the quantity of seeds and optimally inhibits amyloid formation by both TTRwt and TTRv seeds [4]. However, tissue extracts in the study were entirely derived from explanted or autoptic hearts. Anti-seeding therapies should be evaluated in earlier disease stage to avoid potential confounders.

In a *Drosophila* model carrying the V30M TTR mutation, ATTR amyloid accumulation in the thoracic adipose tissue and brain glia caused motor defects. The administration of food mixed with TabFH2 solidified into soft edible gels resulted in the improvement of many locomotor parameters such as mean velocity and travelled distance, compared with control group of treated engineered flies expressing the less severe double mutation pV14N/pV16E (3.8 mm/s vs 2.7 mm/s, *p* = 0.029 and 262 mm vs 143 mm, *p* = 0.005, after 17 days of treatment) [108]. Moreover, Western blotting of the head of flies treated with TabFH2 showed a significant reduction of insoluble ATTR. Two TabFH2 concentrations (100 µM and 300 µM) were considered, with administration of 33.65 nl/h until death, and the results revealed a dose-dependent decrease of amyloid TTR and improvement of locomotor function [108]. Further evaluation of TabFH2 and other anti-seeding strategies is therefore needed in other models and, eventually, humans. Anti-TTR antibodies designed to specifically bind to amyloid fibrils and seeds might represent an effective solution to hinder amyloid seeded-propagation [109]. Peptides have lower manufacturing costs than monoclonal antibodies. Moreover, peptides are smaller and less immunogenic than recombinant antibodies and bigger proteins, but are poorly permeable in tissues because of their hydrophilicity [110]. Peptides have also a limited stability and availability due to rapid degradation by proteolytic enzymes of the digestive system and plasma, as well as fast clearance from the circulation by the liver and kidneys [111]. Therefore, parenteral administration and multiple doses might be needed to reach an effective peptide concentration in tissues [112]. To overcome these limitations, peptides can be altered to increase their protease resistance by chemical modifications, such as C-terminal amidation, N-terminal acetylation and use of non-natural amino acids or D-amino acids [113, 114]. Furthermore, covalent conjugation with polyethylene glycol (PEG) may improve drug solubility and decrease immunogenicity of peptides [114]. Peptides may also be encapsulated into nanoparticles that confer resistance to degradation and allow more targeted release, thus reducing the amount of seeding inhibitors to be administered [114].

## Conclusions

ATTR amyloidosis is being diagnosed in a growing number of patients, which challenges its classification as a rare disease. The prognosis of patients with ATTR amyloidosis is significantly influenced by the timing of diagnosis. When ATTR amyloidosis is recognized in an early disease stage, treatment may be more effective, resulting in a better survival and quality of life. Conversely, the long-term efficacy of therapies decreases if a strong stimulus to amyloid deposition in tissues persists. In vitro studies revealed the ability of amyloid seeds to promote the conversion of soluble TTR molecules into amyloid. These findings led to consider amyloid seeding as a possible mechanism of disease progression, enhancing the spread of amyloid TTR fibrils.

The presence of misfolded TTR in the circulation is the expression of a failure of proteostatic mechanisms. Misfolded TTR proteins must then reach critical local concentration to trigger fibril formation, in conjunction with local factors that modulate aggregation and oligomer formation, including endoproteases. Furthermore, the sonication of ATTR fibrils greatly enhances their seeding capacity by producing fibril fragments, although the relevance of seeds produced by proteolytic degradation in vivo is unclear and probably not very relevant, given that amyloid fibres do not elicit an activation of tissue proteases. The role of proteolytic degradation of TTR for the formation of seeds and disease progression deserves further investigation.

Current therapies for ATTR amyloidosis include interventions at different stages of the amyloidogenic cascade, but the TTR stabilizer tafamidis is the only recommended strategy for the treatment of patients with ATTR cardiomyopathy, with reduced long-term efficacy in advanced disease stages, possibly due to amyloid seeded-propagation, as hypothesized by Saelices et al. [4]. Further studies are needed to better understand the conditions promoting the formation and multiplication of seeds under physiological or near-physiological conditions in vivo, as well as elucidate if all non-native forms of amyloid precursors behave as seeds, and if there are any size, conformation or concentration requirements needed to behave as seeds. A relevant gap indeed still exists between in vivo and in vitro models of amyloidogenesis as the biological complexity of the disease in living organisms cannot be faithfully reproduced in the latter. Moreover, revealing the mechanistic details of the proteolysis-mediated misfolding of TTR is crucial, and characterization of the TTR fragments produced in this process may help elucidate the interplay with amyloid seeds [115]. Future studies should also consider the potential cytotoxic effects caused by the stabilization of small amyloid seeds. Additionally, the pathological cascade may begin months or years before the appearance of the first clinical symptoms, suggesting that seeds in tissues might potentially be used as biomarkers for the early stages of disease. Despite this, it is unknown whether the seeds remain unchanged as the disease progresses or if there are differences between early- and long-standing disease seeds. Finally, the inhibition of amyloid aggregation by anti-seeding peptides such as TabFH2 may block amyloid deposition within tissues in vivo, thereby impairing the self-association and subsequent polymerization of fibrils. Furthermore, treating asymptomatic carriers of *TTR* mutations associated with a high risk of progression may potentially postpone the onset of the severe form of the disease, or partially prevent amyloid deposition.

In conclusion, although peptides are generally considered poor drug candidates due to their scarce stability in vivo and low oral bioavailability, the use of nanoparticles with protease inhibitors and the alteration with chemical modifications may increase the pharmacological potential of anti-seeding peptides. The synergistic combination of TTR stabilizers or gene silencing therapies and anti-seeding inhibitors may represent a novel approach. Stabilizing molecules such as tafamidis can efficiently delay the tetramer dissociation, while seeding inhibitors such as TabFH2 may bind to preformed seeds, thus halting seed propagation. This combined strategy might be considered in preclinical and then possibly clinical studies.
